# Extending Viability of *Bifidobacterium longum* in Chitosan-Coated Alginate Microcapsules Using Emulsification and Internal Gelation Encapsulation Technology

**DOI:** 10.3389/fmicb.2019.01389

**Published:** 2019-06-28

**Authors:** Rui Ji, Jiahui Wu, Junliang Zhang, Tao Wang, Xudong Zhang, Lei Shao, Daijie Chen, Jian Wang

**Affiliations:** ^1^School of Pharmacy, Shanghai Jiao Tong University, Shanghai, China; ^2^Shanghai Institute of Pharmaceutical Industry, China State Institute of Pharmaceutical Industry, Shanghai, China; ^3^College of Engineering, China Pharmaceutical University, Nanjing, China; ^4^Shenzhen Key Laboratory of Marine Bioresource and Eco-environmental Science, Shenzhen University, Shenzhen, China; ^5^College of Life and Environmental Science, Shanghai Normal University, Shanghai, China; ^6^National Pharmaceutical Engineering Research Center, China State Institute of Pharmaceutical Industry, Shanghai, China

**Keywords:** *Bifidobacterium longum*, microencapsulation, sodium alginate, emulsification and internal gelation, chitosan, acid resistant, bile salt resistant

## Abstract

*Bifidobacteria* are considered one of the most important intestinal probiotics because of their significant health impact. However, this ability is usually limited by gastrointestinal fluid and temperature sensitivity. Emulsification and internal gelation is an encapsulation technique with great potential for probiotic protection during storage and the gastrointestinal transit process. This study prepared microcapsules using an emulsification and internal gelation encapsulation method with sodium alginate, chitosan, and *Bifidobacterium longum* as wall material, coating material, and experimental strain, respectively. Optical, scanning electron, and focal microscopes were used to observe the microcapsule surface morphology and internal viable cell distribution, and a laser particle size analyzer and zeta potentiometer were used to evaluate the chitosan-coating characteristics. In addition, microcapsule probiotic viability after storage, heat treatment, and simulated gastrointestinal fluid treatment were examined. Alginate microcapsules and chitosan-coated alginate microcapsules both had balling properties and uniform bacterial distribution. The latter kept its balling properties after freeze-drying, verified by scanning electronic microscopy (SEM), and had a clear external coating, observed by an optical microscope. The particle size of chitosan-coated alginate microcapsules was slightly larger than the uncoated microcapsules. The zeta potential of alginate and chitosan-coated alginate microcapsules was negative and positive, respectively. Heat, acid and bile salt tolerance, and stability tests revealed that the decrease of viable cells in the chitosan-coated alginate microcapsule group was significantly lower than that in uncoated microcapsules. These experimental results indicate that the chitosan-coated alginate microcapsules protect *B. longum* from gastrointestinal fluid and high-temperature conditions.

## Introduction

*Bifidobacterium longum* are an important probiotic bacteria that are able to colonize the human gastrointestinal tract; this species has been added to a variety of dietary supplements, foods, health products, and drugs for regulating intestinal flora, increasing immune function, improving lipid metabolism, and relieving constipation and anti-oxidative action (Jiang et al., 2014; Yeung et al., 2016; Bianchi et al., 2018). *B. longum* is a strictly anaerobic gram-positive bacteria that grows between pH 4.5 and 8.5 and is very sensitive to adverse environmental conditions such as oxygen, humidity, temperature, and stomach acid and bile salts (Lievin et al., 2000). Therefore, the number of viable cells may be greatly reduced by production, storage, and passage through the gastrointestinal tract. However, studies have shown that probiotics must be live and present in sufficient quantity (>10^6^ CFU/g or 10^6^ CFU/ml) to confer a host benefit (Tripathi and Giri, 2014; Ramos et al., 2018). Finding a preparation method to protect *B. longum* from adverse environmental effects is necessary to create a stable and effective probiotic supplement.

Microencapsulation is considered an effective means of entrapping probiotics using natural or synthetic polymers as packaging materials, and encapsulating the material by chemical, physical, or combined physical–chemical methods (Heidebach et al., 2012; Martín et al., 2015). Probiotics can be embedded and isolated from external environments by appropriate microencapsulate preparation for a significantly increased ability to resist acids, bile salts, oxygen, and gastrointestinal conditions (Heidebach et al., 2012). Commonly used wall materials for probiotic microcapsule preparation are sodium alginate and proteins (Cook et al., 2012). Sodium alginate is a natural polysaccharide derived from brown algae or bacteria, and is a safe, non-toxic, biocompatible, and inexpensive material; it is composed of β-D-mannuronic acid (M) and α-L-guluronic acid (G) connected by a 1,4 glycosidic bond. Sodium alginate forms a network structure similar to an “egg box” when Na^+^ on the G unit is exchanged with Ca^2+^, creating cross-links between alginate molecules (Fareez et al., 2015; Huq et al., 2017).

The most commonly used methods for forming probiotic microcapsules using sodium alginate as a wall material are extrusion and emulsification. Compared to the extrusion method, emulsification is milder and simpler and produces microcapsules with a smaller particle size. This does not affect the taste of various products when the microcapsules are added, and the emulsification method is more suitable for large-scale production (Govender et al., 2014; Sarao and Arora, 2017). Emulsification is further divided into the internal and external methods. In the former method, Ca^2+^ is cross-linked with the sodium alginate droplet from the inside to the outside. In the latter, Ca^2+^ diffuses from outside the sodium alginate droplet to the inside. Many studies have shown that alginate microcapsules prepared by the internal method have a more uniform particle size, smoother surface, and higher encapsulation efficiency, while in the external method, agglutination is likely to occur and result in excessive particle size and size dispersion (Zou et al., 2011; Song et al., 2013).

Preparation of alginate microcapsules by the emulsification and internal gelation method is generally composed of the following four steps (Holkem et al., 2016a): (a) Mix sodium alginate solution with insoluble calcium salt and probiotic suspension, (b) add resultant mixture to plant oil containing an emulsifier while constantly stirring to form a water and oil emulsion, (c) add oil-soluble acid into the emulsion to promote Ca^2+^ release from the insoluble calcium salt to form alginate microcapsules, and (d) add washing medium into the emulsion to separate the oil phase from the aqueous phase. The microcapsules in the aqueous phase are collected by centrifugation or filtration techniques.

However, some studies have shown that single alginate material does not provide an acceptable protective effect on probiotic resistance to simulated gastrointestinal fluid (Cook et al., 2011; Haffner et al., 2016). Researchers have found that creating a polymer coating on the alginate microcapsule surface can reduce surface porosity and increase surface hardness and internal stability (Wu et al., 2016). Chitosan, a natural linear cationic polysaccharide consisting of glucosamine and *N*-acetylglucosamine linked by β-1,4 glycosidic bonds, is widely found in crustaceans, hard shells, and insect cuticles. It is a non-toxic, biodegradable material with desirable biocompatibility and biological adhesion properties, and has been widely used in the food and pharmaceutical industries (Lee et al., 2013; Huang et al., 2015). Chitosan molecules contain a large amount of primary amino groups; hence, ionization results in positive charges when the solution pH is lower than pKa 6.5 (Lim et al., 2015). Therefore, chitosan can be electrostatically combined with a negatively charged polymer, such as sodium alginate. The properties of chitosan make it ideal for use as a coating material for protective microencapsulation. Previous studies have shown that the use of chitosan as an alginate microcapsule coating can improve the survival rate of probiotics in storage and gastrointestinal environments (Cook et al., 2011; Zou et al., 2011; Kamalian et al., 2014; Fareez et al., 2015; Ansari et al., 2017; Zhou et al., 2017). Chitosan coating can block the pores of alginate capsules, firmly immobilizing the bacteria within the microcapsules and reducing the probability of probiotic migration (Gaserod et al., 1998; Etchepare et al., 2016). For example, Cook et al. (2011) encapsulated *Bifidobacterium breve* NCIMB 8807 using an external gelation method and chitosan coating and then exposed them to simulated gastric fluid (pH 2.0) for 2 h; *B. breve* only experienced a 2-log reduction, while free cells showed a 9-log reduction. Ding and Shah (2007) tested eight strains of microencapsulated probiotic bacteria for heat tolerance and found that after 30 min of 65°C heat treatment, microencapsulated probiotic bacteria survived with an average loss of only 4.17 log CFU/ml, compared to a 6.74 log CFU/ml loss in free probiotic bacteria.

However, few studies have been conducted on *B. longum* microcapsule preparation combining sodium alginate and chitosan with an emulsification and internal gelation method. Therefore, *B. longum* microcapsules were prepared by emulsification and electrostatic adsorption and then evaluated by particle size and embedding rate. The protective effect of this preparation method on high temperature and simulated gastrointestinal fluids was also evaluated. The goal of this study was to lay the foundation for the industrial application of probiotic microcapsules produced by the emulsification and internal gelation method.

## Materials and Methods

### General Chemicals Used in Encapsulation and Modeled Digestion

Man Rogosa Sharpe (MRS) broth was obtained from Haibo Biotechnology Co. Ltd. (Qingdao, China). Agar, sodium chloride (NaCl), and glycerol were purchased from Sinopharm Chemical Reagent Co. Ltd. (Shanghai, China). For the microencapsulation experiments (including preparation and characterization), sodium alginate, Tween 80, glacial acetic acid, calcium carbonate, sodium L (+)-ascorbate, sodium citrate, sodium bicarbonate, potassium phosphate monobasic, and dipotassium hydrogen phosphate were purchased from Sinopharm Chemical Reagent Co. Ltd. Chitosan hydrochloride and trehalose were purchased from Aladdin Reagent Co. Ltd. (Shanghai, China). Skim milk powder was purchased from NZMP (Auckland, New Zealand). Bean oil was purchased from Yihai Kerry (Shanghai, China). For simulated digestion experiments, sodium hydroxide (NaOH) and concentrated hydrochloric acid (HCl) were purchased from Sinopharm Chemical Reagent Co. Ltd. Trypsin was purchased from Amresco (Dallas, TX, United States). Pepsin and bile salts were purchased from Sigma-Aldrich (St. Louis, MO, United States).

### Bacterial Culture Preparation

*Bifidobacterium longum* strain DD98 was isolated from the feces of a healthy human subject and deposited at the China General Microbiological Culture Collection Center with a deposit number of 16573. Stock solutions were maintained at −80^∘^C (Fermo^TM^ 900, Thermo Scientific, Waltham, MA, United States) in MRS media with 20% glycerol. Bacteria were propagated in MRS liquid medium (300 ml) at a 1.0% inoculation rate for 24 h at 37°C in an anaerobic environment and checked for purity. The anaerobic systems were maintained in an anaerobic box with 4% CO_2_ (Concept 400M, Ruskinn, Bridgend, United Kingdom). Probiotic suspensions were harvested by centrifugation at 4,000 × *g* for 10 min, washed twice with 25 ml of 0.85% NaCl (physiological saline) solution, and then either used directly for free cell survival assessment or subjected to encapsulation.

### Microencapsulation of *Bifidobacteria* Cells

**FIGURE 1:**
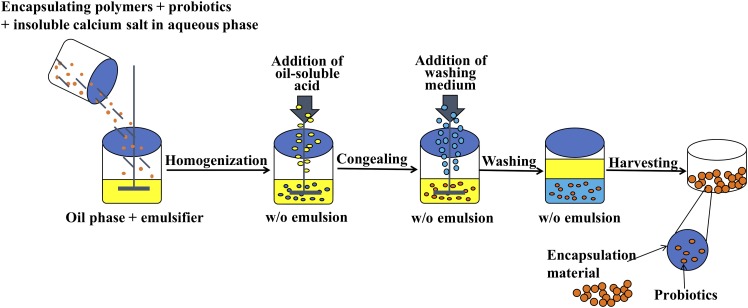
Emulsification and internal gelation method probiotic encapsulation process.

Microcapsule preparation using the emulsification and internal gelation method was based on the methods of Poncelet et al. (1992) with slight modifications. First, 25 ml of 1.5% sodium alginate solution was mixed with 2.5 g of *B. longum* suspension and 0.000625 mol CaCO_3_ powder. The mixture was added dropwise to 100 ml of soybean oil containing 1.0% Tween 80 and stirred constantly at 600 rpm using a mechanical stirrer (RCT, IKA, Baden-Württemberg, Germany) equipped with a 10 × 70 mm magnetic agitator to form an emulsion. The reaction was carried out in a round-bottomed reactor (internal diameter of 90 mm and height of 120 mm). After stirring for 15 min, 25 ml of soybean oil containing 150 μl of glacial acetic acid was added. After 5 min, 1.0% Tween 80 washing medium solution was added while stirring slightly to separate the oil phase from the aqueous phase. Finally, the above mixture was centrifuged (1,500 × *g*, 10 min), and the upper oil phase and the aqueous phase were discarded to obtain the precipitate (microcapsules). The microcapsules were washed twice with 50 ml of washing medium to remove the residual oil phase and then washed once with 25 ml of 0.85% NaCl solution to obtain *B. longum* alginate microcapsules (Alg-Bl) (Figure 1).

The microcapsule coating method was performed using the methods of Zhang et al. (2005) with slight modifications. A 0.4% w/v aqueous chitosan solution was prepared. Chitosan (0.4 g) was dissolved in 90 ml of distilled water and 0.4 ml of glacial acetic acid. The pH was adjusted to 6.0 using NaOH, and the total volume was adjusted to 100 ml using purified water. The solution was autoclaved and filtered to remove undissolved solids. Subsequently, 5 g of the newly prepared alginate microcapsules was suspended in 20 ml of purified water and then submerged in the chitosan solution while stirring at 100 rpm for 10 min to provide a coating by electrostatic attraction. After the coating was completed, the precipitate was collected by centrifugation (1,500 × *g*, 10 min). Chitosan-coated alginate microcapsules (Ch-Alg-BL) were washed twice with 25 ml of sterile 0.85% NaCl solution.

### Characterization of Alginate and Chitosan-Coated Alginate Microcapsules

#### Encapsulation Yield Determination

To determine the viable encapsulated bacterial counts, 1 ml of alginate microcapsules was resuspended in 9 ml of 0.1 mol/L phosphate buffer, 1 ml of chitosan-coated microcapsules was re-suspended in a 9-ml mixed solution of 0.2 mol/L NaHCO_3_ and 0.06 mol/L trisodium citrate, and each was vortexed using a vortex mixer (Vortex-genie 2, Scientific Industries, Austin, TX, United States) for approximately 1 min. After the microcapsules were completely dissolved, series dilutions (10^–1^ to 10^–7^) were prepared with physiological saline, and 100 μl of each dilution was coated onto sterilized and cooled MRS agar plates in triplicate. Dilutions with 30–300 visible CFU per plate were used to enumerate viable *B. longum* cells (CFU/g).

$$\mathrm{Encapsulation}\,\mathrm{yield}\,\left(\mathrm{EY}\right)=\frac{N}{N_{0}}\times 100$$

where *N* was the number of viable entrapped cells released from the microcapsules and *N*_0_ was the number of free cells added to the biopolymer mix during microcapsule production.

#### Particle Size Distribution

The prepared alginate and chitosan-coated alginate capsules were pipetted and added dropwise to the sample cell. The particle size was determined using a laser diffraction particle size analysis system (LA-920, HORIBA, Kyoto, Japan) using water as the dispersion medium. The SPAN value was used to indicate the particle size dispersion.

$$\mathrm{SPAN}=\frac{\left[D\,\left(v,90\right)-D\,\left(v,10\right)\right]}{D\,\left(v,50\right)}$$

where *D* (*v*, 90), *D* (*v*, 50), and *D* (*v*, 10) represented the average particle diameter of the microcapsules at 90, 50, and 10% cumulative volume, respectively.

#### Zeta Potential Measurement

The prepared alginate and chitosan-coated alginate capsules were dispersed in purified water and then added to a Zeta potentiometer sample cell (Zetasizer Nano ZSP, Malvern Panalytical, Malvern, United Kingdom). The sample solution was left to settle naturally for 5 min and then was placed on the Zeta potentiometer for potential measurement.

#### Morphological Observation

A drop of the dispersed microcapsule solution was placed on a slide using a glass rod. Microcapsule morphology was observed and photographed with an optical microscope (Eclipse E200, Nikon, Kyoto, Japan).

#### Scanning Electronic Microscopy

A layer of double-sided tape was attached to the scanning electron microscope sample stage, and the alginate and chitosan-coated alginate capsule powder samples were sprayed onto the one-sided tape and then sputtered with gold and observed on a low-vacuum high-resolution scanning electron microscope (TM3030Plus, Hitachi, Kyoto, Japan).

#### Confocal Scanning Laser Microscopy

A 0.1-g sample of alginate capsules, chitosan-coated alginate capsules, and free *B. longum* were each dispersed in 500 μl of purified water, and then 20 μl of 0.1% acridine orange solution was added and then incubated at 37°C in a shaking incubator (ZWY-240, ZHCHENG, Shanghai, China) at 200 rpm for 30 min. The supernatant was removed by centrifugation, and the precipitate was washed three times with purified water. After centrifugation, a drop of each sample was placed onto glass slides and observed using a confocal laser-scanning microscope (TCS SP8 STED, Leica Microsystems, Wetzlar, Germany) at excitation and emission wavelengths of 488 and 580 nm, respectively.

#### Survival of Free and Microencapsulated *B. longum* Under Heat Treatments

To measure the tolerance of encapsulated *B. longum* to heat treatment (55, 60, and 65°C for 30 min), alginate capsules, chitosan-coated alginate capsules, and free *B. longum* (1 ml) were each added to 9 ml of preheated sterile distilled water (pH 6.54) as a suspending medium, and this method was described by Mandal et al. (2006) and Abbaszadeh et al. (2014). The content was cooled to room temperature after heat treatment and viable cell count as described in the section Encapsulation Yield Determination.

#### Survival of Free and Microencapsulated *B. longum* Under Long-Term Storage

Alginate capsules, chitosan-coated alginate capsules, and free *B. longum* were uniformly mixed with lyoprotectant in a 1:1 mass ratio for vacuum freeze drying in a freeze dryer (LGJ-22D, Sihuan, Beijing, China). The formulation of the lyoprotectant was 20% trehalose, 10% skim milk powder, 2% vitamin C sodium, and 68% water (w/w). Samples were pre-frozen for 3 h at −70°C and then freeze-dried for 24 h at 15°C. After lyophilization, samples were stored at 4 and 25°C, and the viable cell count was determined at 0, 15, 30, 60, 90, and 180 days.

### Survival of Free and Microencapsulated *B. longum* in Simulated Gastrointestinal Fluid

The viability of free and encapsulated probiotics in simulated gastric and intestinal fluid was determined using the methods described by Kamalian et al. (2014) and Yasmin et al. (2018), respectively, with some modifications. Simulated gastric fluid was prepared by adjusting the pH of a 0.5% NaCl solution to 2.50 ± 0.02 using concentrated HCl, and then 3 g/L pepsin was added. Simulated intestinal fluid was prepared by adding 6.8 g of dipotassium hydrogen phosphate to 250 ml of distilled water and then adding 77 ml of 0.2 mol/L NaOH solution, 10 g of trypsin, and 1 g of bovine bile salt. The pH was adjusted to 6.80 ± 0.02 using 0.2 mol/L NaOH solution, and then the volume was adjusted to 1 L using distilled water. Finally, the solutions were sterilized using 0.22-μm filters. Alginate capsules, chitosan-coated alginate capsules, and free *B. longum* (1 ml) were added to 9 ml of simulated gastric or intestinal fluid and kept at 37°C in a shaking incubator (ZWY-240, ZHCHENG, Shanghai, China) at 100 rpm. A sample from each group (0.1 ml) was taken at 0, 5, 30, 60, and 120 min for a viable cell count. In addition, the survival of encapsulated and free *B. longum* in continuous simulated gastrointestinal fluid was also investigated; we collected capsules of *B. longum* and then transferred them to simulated intestinal fluid for 2 h after incubating them in simulated gastric fluid for 2 h, and a sample from each group (0.1 ml) was taken at 30, 60, 120, 180, and 240 min for a viable cell count.

### Statistical Analysis

The data were represented as the mean ± standard deviation with *n* ≥ 3 (except for the label) and processed by GraphPad Prism7 and SPSS 23.0 statistical analysis software. One-way ANOVA followed by Tukey’s test was used to compare the mean difference between more than two groups, and the independent-samples *t* test was used to compare the mean difference among two groups. *p* < 0.05 was considered to be significantly different.

## Results

### Encapsulation Yield of Microcapsules

The encapsulation yield was an important index to evaluate the microcapsule embedding effect. Free cell suspension bacterial density was approximately 10^9^–10^10^ CFU/g. The encapsulation yield of alginate microcapsules and chitosan-coated microcapsules reached 95 ± 2.5% and 90 ± 3.4%, respectively. These results indicate that *B. longum* microcapsule preparation by emulsification and internal gelation and electrostatic adsorption methods efficiently maintained high probiotic activity.

### The Analysis of Microcapsule Particle Size and Electrical Properties

The mean diameter of alginate microcapsules was approximately 160 μm, and chitosan-coated alginate microcapsules were approximately 190 μm (Table 1). Electrostatic interaction between alginate and chitosan formed a polyelectrolyte membrane on the surface of alginate capsules; therefore, the particle size of coated capsules was slightly larger than uncoated. Additionally, alginate capsules were slowly added to the chitosan solution; thus, the reaction occurred over time during coating; this contributed to the particle size dispersion of coated capsules being slightly larger than the uncoated capsules.

**TABLE 1:** Particle size and surface potential of alginate microcapsules (Alg-Bl) and chitosan-coated alginate microcapsules (Ch-Alg-Bl).

	**Alg-Bl**	**Ch-Alg-Bl**
Mean diameter (μm)	$154.3\pm 3.5{}^{a}$	$190.1\pm 2.4{}^{b}$
Span factor	$1.15\pm 0.06{}^{a}$	$1.45\pm 0.03{}^{b}$
Zeta potential (mv)	$-13.3\pm 0.7{}^{a}$	$4.2\pm 0.3{}^{b}$

*Values are shown as the mean ± standard deviation. Values within each row followed by the same lowercase letters are not significantly different (*p* > 0.05).*

The zeta potential of alginate and chitosan-coated alginate microcapsules was negative and positive, respectively, which was consistent with the theoretical prediction that positively charged chitosan would adhere to the negatively charged sodium alginate surface.

### Optical Microscopy and Scanning Electronic Microscopy Characterization

**FIGURE 2:**
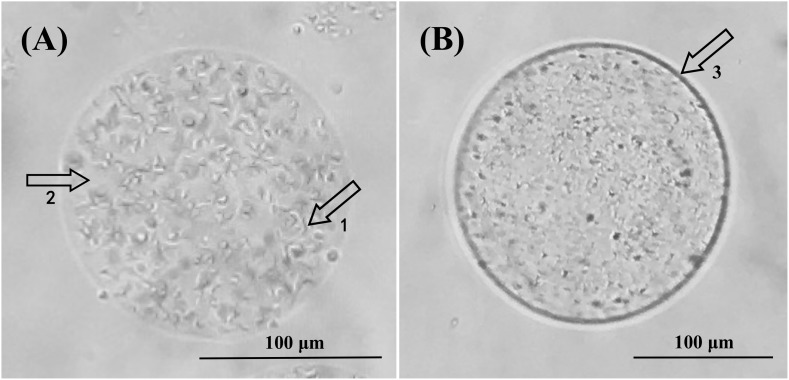
Optical microscope views of alginate microcapsules **(A)** and chitosan-coated alginate microcapsules **(B)**. **(A)** At 100× magnification, 1 and 2 indicate *Bifidobacterium longum* and calcium alginate, respectively. **(B)** At 100× magnification, 3 shows the chitosan coating layer.

**FIGURE 3:**
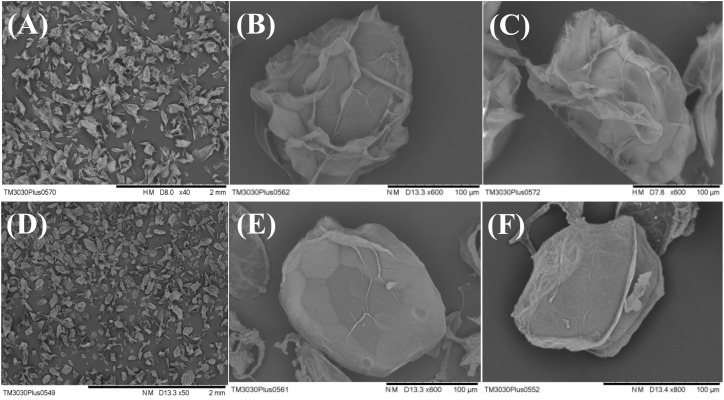
Scanning electron micrographs of alginate microcapsules **(A–C)** and chitosan-coated alginate microcapsules **(D–F)**. Samples were freeze-dried before sputter-coating with gold. Chitosan-coated microcapsules have better sphericity than uncoated microcapsules after lyophilization.

In order to further understand the microscopic microcapsule morphology, newly prepared and freeze-dried microcapsules were observed under optical microscopy and scanning electron microscopy, respectively (Figures 2, 3). Figure 2 showed that Alg-Bl and Ch-Alg-Bl both have a regular spherical structure, with the inner core of *B. longum* evenly embedded in the structure; the alginate mesh skeleton structure can also be observed. An obvious chitosan layer was present on the alginate microcapsule surface (Figure 2B). Freeze-dried microcapsule surfaces became heavily wrinkled and lost their spherical shape, forming an irregular structure (Figure 3). Sodium alginate is a colloid and swells in an aqueous solution to maintain a spherical structure; dehydration during lyophilization resulted in a loss of ability to maintain that spherical structure. Figure 3 also shows that chitosan-coated microcapsules had better sphericity than uncoated microcapsules after lyophilization.

### Confocal Micrograph Characterization

**FIGURE 4:**
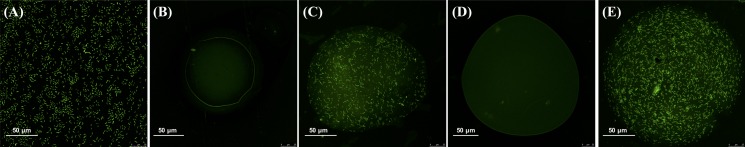
Confocal micrographs of panel **(A)** *Bifidobacterium longum*, **(B)** alginate microcapsule without *B. longum*, **(C)** alginate microcapsule with *B. longum*, **(D)** chitosan-coated alginate microcapsule without *B. longum*, and **(E)** chitosan-coated alginate microcapsule with *B. longum*. All samples were stained with acridine orange fluorescent dye to enable DNA detection.

In order to better understand *B. longum* distribution, acridine orange was used as a fluorescent dye to verify distribution within the microcapsules by laser confocal technique. Staining results were clear and representative; the fluorescence intensity of microcapsules before and after coating was strong (Figure 4). *B. longum* cells were clearly visible and evenly distributed in the capsule. The fluorescence intensity of the empty microcapsules was significantly lower, and no bacteria were seen (Figures 4B,D).

### Survival of Free and Microencapsulated *B. longum* During Heat Treatment and Long-Term Storage

**FIGURE 5:**
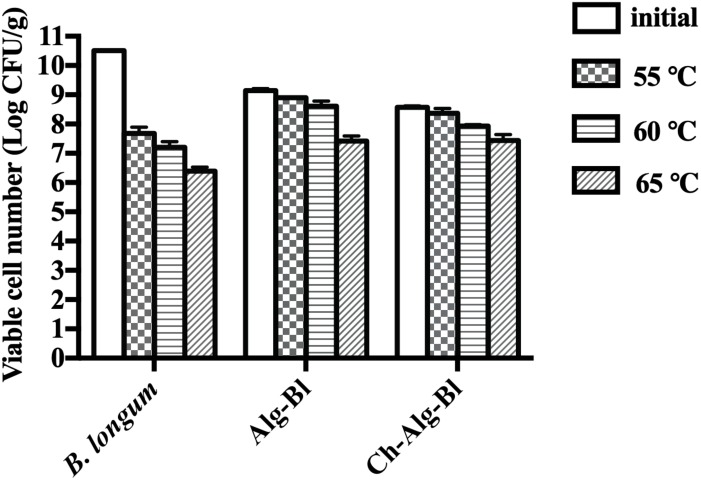
Viable bacterial count of *Bifidobacterium longum*, Alg-Bl, and Ch-Alg-Bl after heat treatment at 55, 60, and 65°C for 30 min.

**FIGURE 6:**
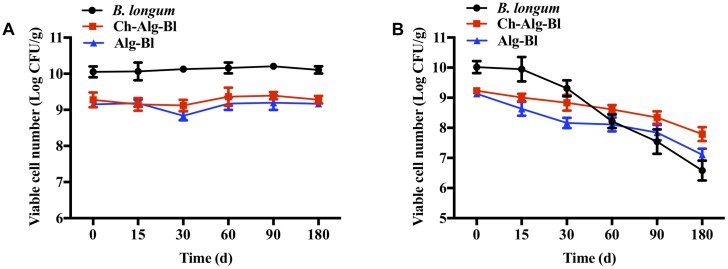
The storage stability of *Bifidobacterium longum*, Alg-Bl, and Ch-Alg-Bl at 4°C **(A)** and 25°C **(B)**.

Three groups were tested for heat resistance at 55, 60, and 65°C for 30 min (Figure 5). For non-encapsulated *B. longum* exposed to 55, 60, and 65°C, the bacterial count decreased by 2.83, 3.31, and 4.12 log CFU, respectively. The *B. longum* loaded in chitosan-coated/alginate microparticles showed higher heat stability than the free cells. The viability of alginate microcapsules decreased by 0.24, 0.53, and 1.72 log CFU and chitosan-coated microcapsules decreased by 0.20, 0.64, and 1.14 log CFU at 55, 60, and 65°C, respectively. These results indicated that the heat resistance of encapsulated *B. longum* was significantly improved compared to the non-encapsulated *B. longum.* The viable cell change was not obvious over time at 4°C in all three groups (Figure 6A). At 25°C, the viability of free *B. longum* significantly decreased by 3.43 log CFU at 180 days, alginate microcapsules decreased by 2.03 log CFU at 180 days, while chitosan-coated alginate microcapsules decreased by 1.44 log CFU at 180 days (Figure 6B). Based on these results, encapsulated *B. longum* had better storage stability at 25°C than the non-encapsulated *B. longum*.

### Survival of Free and Encapsulated *B. longum* During Exposure to Simulated Gastrointestinal Fluids

Free and encapsulated *B. longum* cells were immersed separately in simulated gastric and intestinal fluids and assessed over time for cell viability. Microencapsulation provided enhanced protection for *B. longum* incubated in simulated stomach and intestinal fluids (Table 2). When exposed to pH 2.5, the viability of free *B. longum* significantly decreased by 3.75 log CFU at 60 min and could not be detected at 120 min, alginate microcapsules decreased by 3.88 log CFU at 30 min and could not be detected at 60 min, while chitosan-coated alginate microcapsules only decreased by 1.27 log CFU at 120 min. Similarly, the viability of free *B. longum* significantly decreased by 5.14 log CFU at 5 min and could not be detected at 15 min, alginate microcapsules decreased by 5.01 log CFU at 15 min and could not be detected at 30 min, while chitosan-coated alginate microcapsules only decreased by 2.68 log CFU at 120 min when exposed to intestinal fluid with bile salt (1%). After continuous simulated gastrointestinal fluid treatment, the viability of free *B. longum* and alginate microcapsules significantly decreased and could not be detected, consistent with the results in Table 2, while chitosan-coated alginate microcapsules only decreased by 2.76 and 3.91 log CFU when treated at 180 and 240 min, respectively. These results showed that chitosan-coated microcapsules protected *B. longum* from gastric acid and bile salt injury.

**TABLE 2:** Simulated digestion of free and encapsulated *Bifidobacterium longum.*

	**Free cells**		**Alg-Bl**		**Ch-Alg-Bl**	
**pH**	**Gastric**	**Intestinal**	**Gastric**	**Intestinal**	**Gastric**	**Intestinal**
	2.50±0.02	6.80±0.02	2.50±0.02	6.80±0.02	2.50±0.02	6.80±0.02

**Time (min)**	**Log CFU/g ± SEM**					

0	$9.30\pm 0.08{}^{\mathrm{aA}}$	$9.30\pm 0.08{}^{\mathrm{aA}}$	$9.08\pm 0.18{}^{\mathrm{aA}}$	$9.08\pm 0.18{}^{\mathrm{aA}}$	$9.11\pm 0.06{}^{\mathrm{aA}}$	$9.11\pm 0.06{}^{\mathrm{aA}}$
5	$8.83\pm 0.53{}^{a}$	$4.16\pm 0.10{}^{b}$	$8.56\pm 0.21{}^{a}$	$6.09\pm 0.26{}^{b}$	$8.99\pm 0.06{}^{\mathrm{ab}}$	$8.20\pm 0.53{}^{b}$
15	$8.66\pm 0.13{}^{a}$	–	$6.95\pm 0.41{}^{b}$	$4.07\pm 0.17{}^{c}$	$8.69\pm 0.41{}^{\mathrm{ab}}$	$7.27\pm 0.06{}^{\mathrm{cf}}$
30	$7.31\pm 0.19{}^{b}$	–	$5.20\pm 0.38{}^{c}$	–	$8.59\pm 0.36{}^{\mathrm{ac}}$	$7.45\pm 0.29{}^{\mathrm{bc}}$
60	$5.55\pm 0.19{}^{c}$	–	–	–	$8.34\pm 0.27{}^{\mathrm{bc}}$	$6.94\pm 0.44{}^{\mathrm{ce}}$
120	–	–	–	–	$7.84\pm 0.26{}^{c}$	$6.43\pm 0.13{}^{\mathrm{def}}$

*Values are shown as the mean ± standard deviation. Means within each column followed by the same lowercase letters are not significantly different (*p* > 0.05). Means within each row followed by the same uppercase letters are not significantly different (*p* > 0.05).*

## Discussion

In our experiment, *B. longum* microcapsules were prepared by the emulsification and internal gelation method using chitosan and alginate as the primary materials. Chitosan coating produced smooth, negatively charged microspheres with a mean size of 190 μm, similar to previous reports (Holkem et al., 2016b; Yeung et al., 2016). In addition, fluorescence microscopy demonstrated that *B. longum* were uniformly dispersed inside the sphere, and the cells were clearly visible. Similar results were obtained in previous studies (Song et al., 2013; Prisco et al., 2015), but the dyeing method used in our study has additional beneficial properties. We used acridine orange as a fluorescent pigment that can bind to DNA and RNA in different quantities and can fluoresce different colors. The dye is membrane permeable and can penetrate cell membranes to stain nuclear DNA and RNA (Fan et al., 2006). Therefore, under a fluorescence microscope, acridine orange can pass through the normal cell membrane, making the nucleus fluoresce green or yellow-green, and we can clearly observe the bacteria in the microcapsules.

More interestingly, the tests performed in our study demonstrated that chitosan-coated microcapsules prepared by the emulsification and internal gelation method improved *B. longum* stability at 25 and 60°C, and significantly protected *B. longum* from gastric acid and bile salt injury. These findings differed from the results of Yeung et al. (2016), where the authors used an extrusion method and chitosan coating to encapsulate *B. longum* and then exposed the microcapsules to simulated gastric fluid (pH 2.53–2.57) and intestinal fluid (0.83% bile salt); the protective effect was not obvious in this study. Neither free cells nor encapsulated cells were detected after 10 min in gastric fluid or 5 min in intestinal fluid, respectively. This difference may have been caused by the specific probiotic strains, or the preparation method. In addition, we found the viable count in Alg-Bl microcapsules to decrease when exposure to simulated gastrointestinal fluids was unexpected. We hypothesized that this might be due to the substitution of calcium alginate into alginic acid when it reacts with a strong acid, thus further lowering the pH of the reaction system. We did a verification. First, we washed the three samples with physiological saline to maintain the initial pH of about 7.0 and then added it to the gastric acid solution with pH 2.5 according to the amount of the reaction system. We found that the pH of the two groups in Ch-Alg-Bl and *B. longum* was maintained at around 3.0, while the pH of the Alg-Bl group was around 2.3. This result also confirmed our hypothesis. In order to ensure the parallelism of the results, we only adjusted the initial pH of the three samples. Results show that chitosan-coated microcapsules have better acid resistance, which can be illustrated by the fact that the chitosan adsorbs on the surface of sodium alginate, thus making up for the porous defects of sodium alginate, so that the cells are firmly fixed in the microcapsules and the effective contact area with gastric acid and bile salts is small.

Attention should be paid to the pH value of the gastric juice and the content of the bile salts when comparing the results of gastric juice and intestinal fluid experiments, as these differ between studies (Cook et al., 2011; Haffner et al., 2016). Finding a suitable preparation method and material for specific probiotic strains is highly important to increase bacterial viability when exposed to digestive juices or heat.

The emulsification and internal gelation method has many potential advantages, in addition to the benefits mentioned in the Introduction. It is easy to scale up and has fewer restrictions on preparation devices (Chen and Chen, 2007; Burgain et al., 2011). Additionally, microcapsules prepared by this method can be easily added to many products, resulting in items that are convenient for consumers to take, are easy to transport, and do not require refrigeration. This approach allows for more extensive applications of *B. longum* in food and medicine. However, this approach has some disadvantages. The plant oil used to create the emulsion is not easily sterilized and is prone to waste (Gbassi and Vandamme, 2012). However, there are many oil recovery technologies available, and it is believed that costs and pollution can be minimized (Sağlık et al., 2015; Photaworn et al., 2017). Attention should be paid to the combination of prebiotics and microcapsules, as well as the application of multi-layer embedding technology; these new methods will bring additional benefits for future probiotic applications (Krasaekoopt et al., 2004; Etchepare et al., 2016; Zhou et al., 2017).

## Data Availability

All datasets generated for this study are included in the manuscript and/or the Supplementary Files.

## Author Contributions

RJ, JWa, and DC designed the experiments. RJ and JWu conducted the laboratory experiments, analyzed the data, and drafted the manuscript. JZ, TW, and XZ assisted with the experiments and data analyses, and contributed to the manuscript. DC supervised the execution of the experimental plan, analyzed the data, and critically reviewed the final version of the manuscript. All authors read and approved the manuscript prior to submission.

## Conflict of Interest Statement

The authors declare that the research was conducted in the absence of any commercial or financial relationships that could be construed as a potential conflict of interest.
